# Dynamic FDG PET/CT on bladder paraganglioma: A case report

**DOI:** 10.3389/fmed.2022.1002663

**Published:** 2022-10-10

**Authors:** Makoto Taninokuchi Tomassoni, Arrigo Cattabriga, Caterina Gaudiano, Federica Ciccarese, Beniamino Corcioni, Lorenzo Bianchi, Riccardo Schiavina, Eugenio Brunocilla, Rita Golfieri

**Affiliations:** ^1^Department of Radiology, IRCCS Azienda Ospedaliero Universitaria di Bologna, Bologna, Italy; ^2^Division of Urology, IRCCS, Azienda Ospedaliero Universitaria di Bologna, Bologna, Italy; ^3^University of Bologna, Bologna, Italy

**Keywords:** bladder paraganglioma, bladder cancer, FDG PET/CT, nuclear medicine, radiology

## Abstract

Paraganglioma (PGL) is characterized by equivocal clinical manifestations and arriving to a suspicion might be challenging. Nevertheless, diagnostic imaging and nuclear medicine are a fundamental part of the diagnosis and management of this particular neuroendocrine tumor (NET). We herein report a rare case of bladder paraganglioma with unusual onset and typical PET/CT characteristics that led to its recognition.

## Introduction

Bladder paraganglioma (BPG) is a neuroendocrine tumor originating from the chromaffin tissue of the bladder wall, more specifically from the sympathetic nervous system embedded in the muscle layer. Belonging to the category of extra-adrenal pheochromocytoma, it accounts for 0.06% of all bladder tumors and 1% of overall paragangliomas (1–3) and it's therefore considered a very rare yet severe condition as it could lead to hypertensive crisis during handling and mobilization (4).

More than one-third of BPGs are malignant and may be either non-functioning or functioning, the latter usually presenting with micturitional attacks, consisting of a variety of symptoms during urination, such as palpitations, headache, fainting and visual disturbances. Nevertheless, almost 30% of patients affected by BPG do not present with any specific symptoms. Consequently, medical imaging plays an important role in order to achieve an early diagnosis (5–7).

Ultrasound (US), computed tomography (CT), and magnetic resonance imaging (MRI) are important to identify, localize and morphologically describe the tumor. BPGs are submucosal hypervascular lesions that in up to 40% of cases are located in the bladder dome (8–10). Cystoscopy is substantially limited, mostly being able to exclude mucosal involvement, typical of urothelial lesions, whilst biopsy is contraindicated since could unleash a hypertensive episode in patients without the correct medical treatment (11).

Lastly, nuclear medicine studies are essential to evaluate the functional pattern of the lesion and to exclude metastases. ^123^I-MIBG is a guanidine analog similar to noradrenaline that, when administered, is stored in the adrenergic cells of paragangliomas that are equipped with noradrenaline transporters and can be used in nuclear medicine exams. On the other hand, BPGs express somatostatin receptors, that bounds to ^68^Ga-DOTANOC, a somatostatin analog used in nuclear medicine imaging (12, 13). ^18^F-FDOPA is an amino acid analog that, once decarboxylated to ^18^F-dopamine, is stored in secretory vesicles of PGL cells (14).

While both ^123^I-MIBG SPECT/CT and ^68^Ga-DOTANOC PET/CT are routinely used for the diagnosis of NETs and, in particular, paragangliomas, it has been demonstrated that ^68^Ga-DOTANOC PET/CT is superior to ^123^I-MIBG SPECT/CT in providing valuable information for staging extra-adrenal PGL (15). Therefore, ^68^Ga-DOTANOC PET/CT should be considered as the first-line investigation study for nuclear medicine diagnosis of PGLs (16).

Nevertheless, in not-specialized centers, this particular radionuclide might not be available.

In this case, dynamic FDG PET/CT could be used in addition to other clinical and imaging data in order to reach a correct diagnosis, as PGLs show a typical pattern with early uptake of FDG and wash-out in the delayed 1-h acquisition phase, taking into account the risk of false negative results (17, 18).

We herein present the case of a patient affected by BPG with non-specific symptoms that were further investigated with radiological and nuclear medicine imaging.

## Case report

A 59-years-old male with no pathological history presented to the Emergency Room with 12-h acute urinary retention. Laboratory tests were normal, except for a small reduction of red blood cells (4.2 million/ml), hematocrit (38.9%) and hemoglobin 13.5 g/dl.

**Figure 1:**
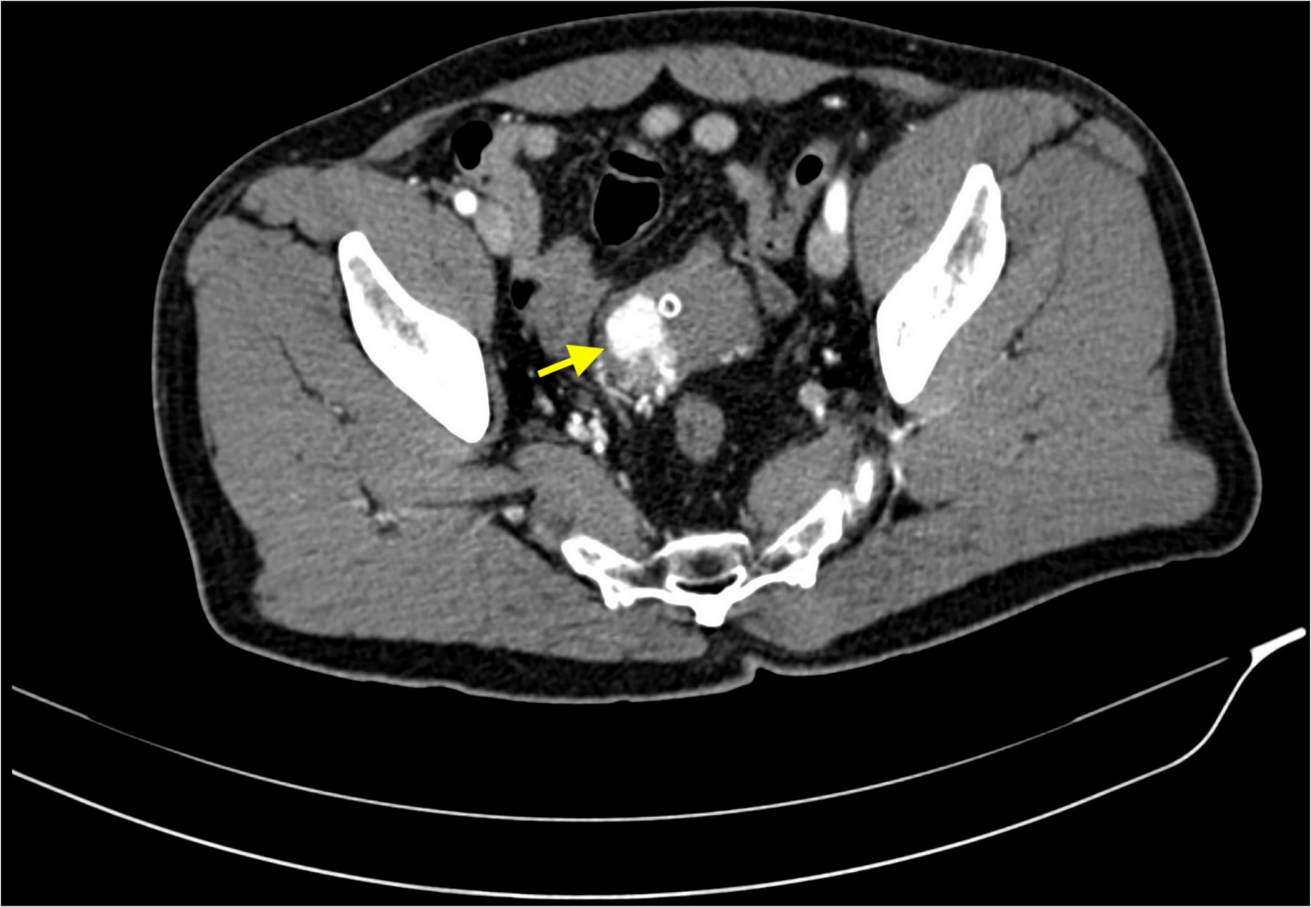
CT scan acquired in arterial phase, after intravenous administration of iodinated contrast media. This image shows a highly vascular lesion within the right wall of the bladder dome (yellow arrow). Some small feeding arteries are appreciable too. The bladder is empty due to urinary catheterization *via* Foley catheter.

After Foley catheter positioning, it was documented significative hematuria with clots emission. The patient was therefore admitted to the urology department of our hospital and a CT Urography was performed, demonstrating a significantly contrast-enhanced bladder nodule fed by thin arterial vessels originating from both left and right hypogastric arteries (Figure 1). It was concluded that these findings were suggestive both of a neoplasm or a vascular arteriovenous malformation. Consequently, further examinations were needed.

The patient underwent abdominal Ultrasonography, to determine whether the lesion was a solid neoplasm (hyperechogenic) or a vascular formation (hypo-anechogenic). The US documented a solid lesion protruding into the bladder wall, characterized by an intense Color-Doppler signal, suggestive of solid hyper-vascular neoplasm. On the same day, a cystoscopy was performed, showing a posterolateral voluminous protruding mass, without any mucosal involvement; therefore, the appearance was not typical for urothelial carcinoma.

**Figure 2:**
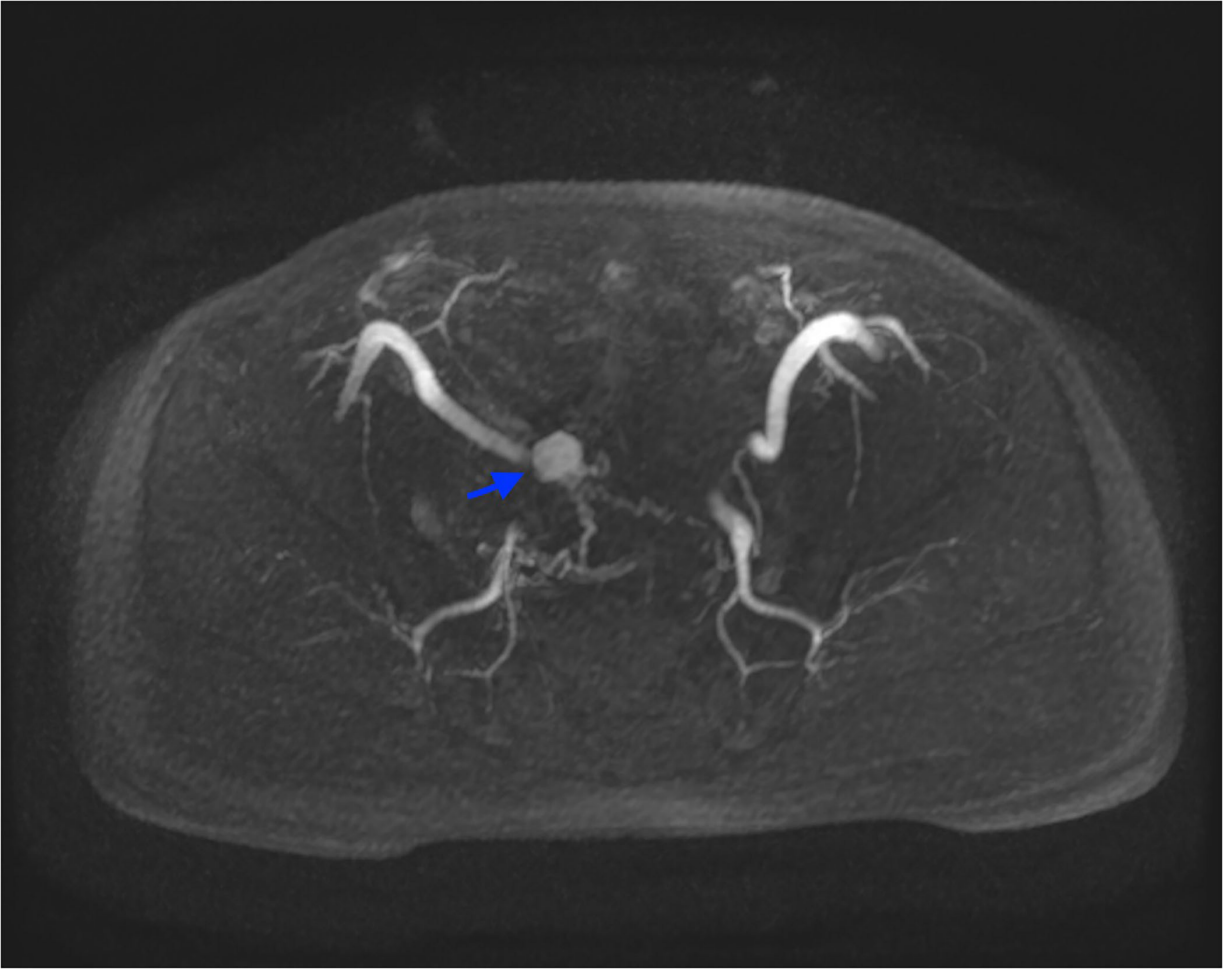
MRI angiography, axial LAVA sequence acquired in arterial phase after administration of intravenous contrast media, with Maximum Intensity Projection reformatting. This image depicts the highly vascularized lesion and its feeding vessels (blue arrow).

The findings of CT-scan and US, showing a solid hyper-vascular lesion of the bladder dome, suggested the suspicion of BPG. The patient subsequently underwent MRI angiography, that clearly depicted the lesion and its multiple afferent feeding arteries, confirming the findings described on CT-scan (Figure 2).

**Figure 3:**
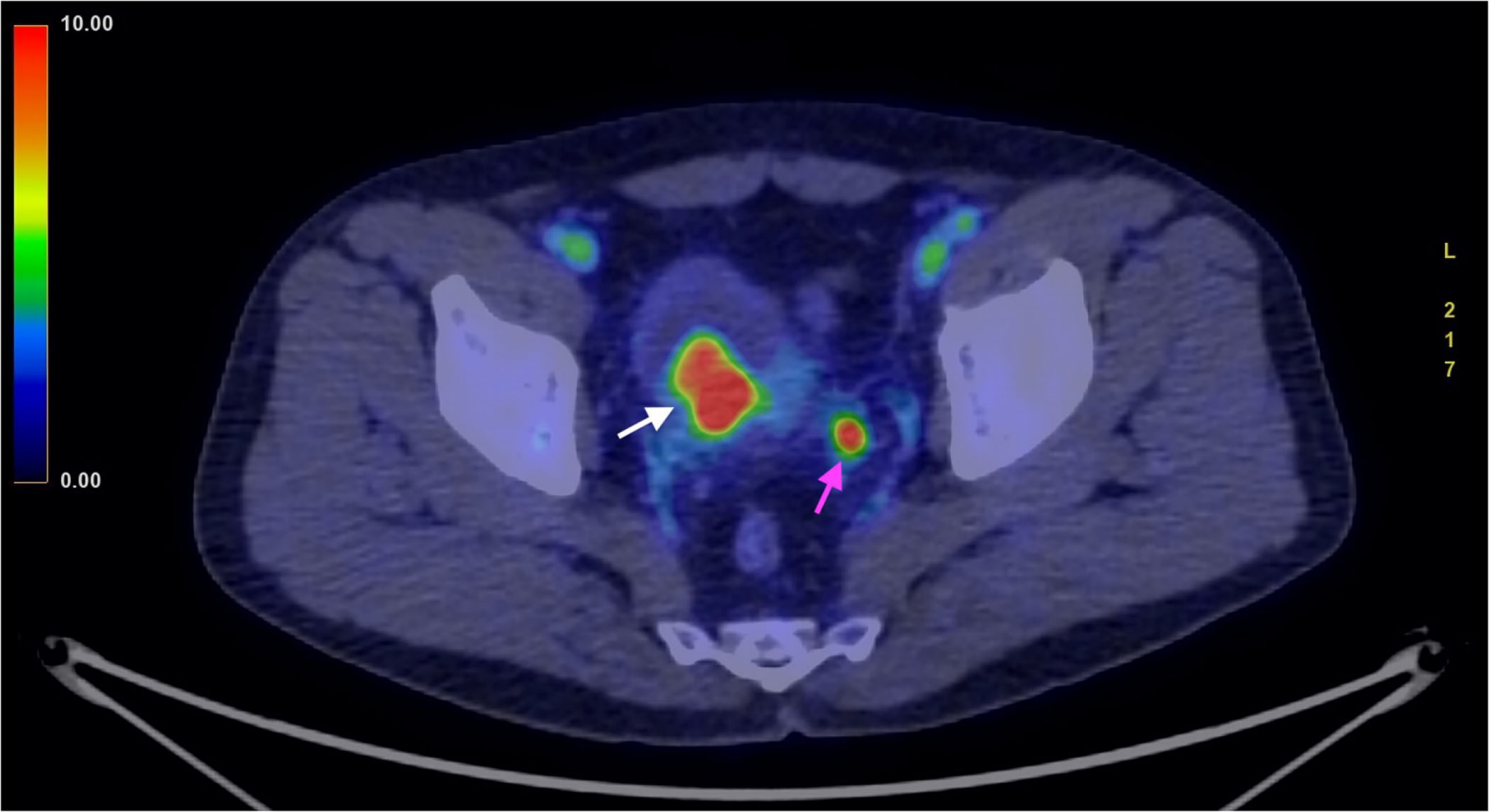
FDG PET/CT (image fusion technique) acquired in an early phase, 3 min after administration of the ^18^FDG. The image shows intense uptake of the ^18^FDG by the tumor (white arrow). The smaller high uptake spot on the left corresponds to physiological collection of urine within the ureter (purple arrow).

**Figure 4:**
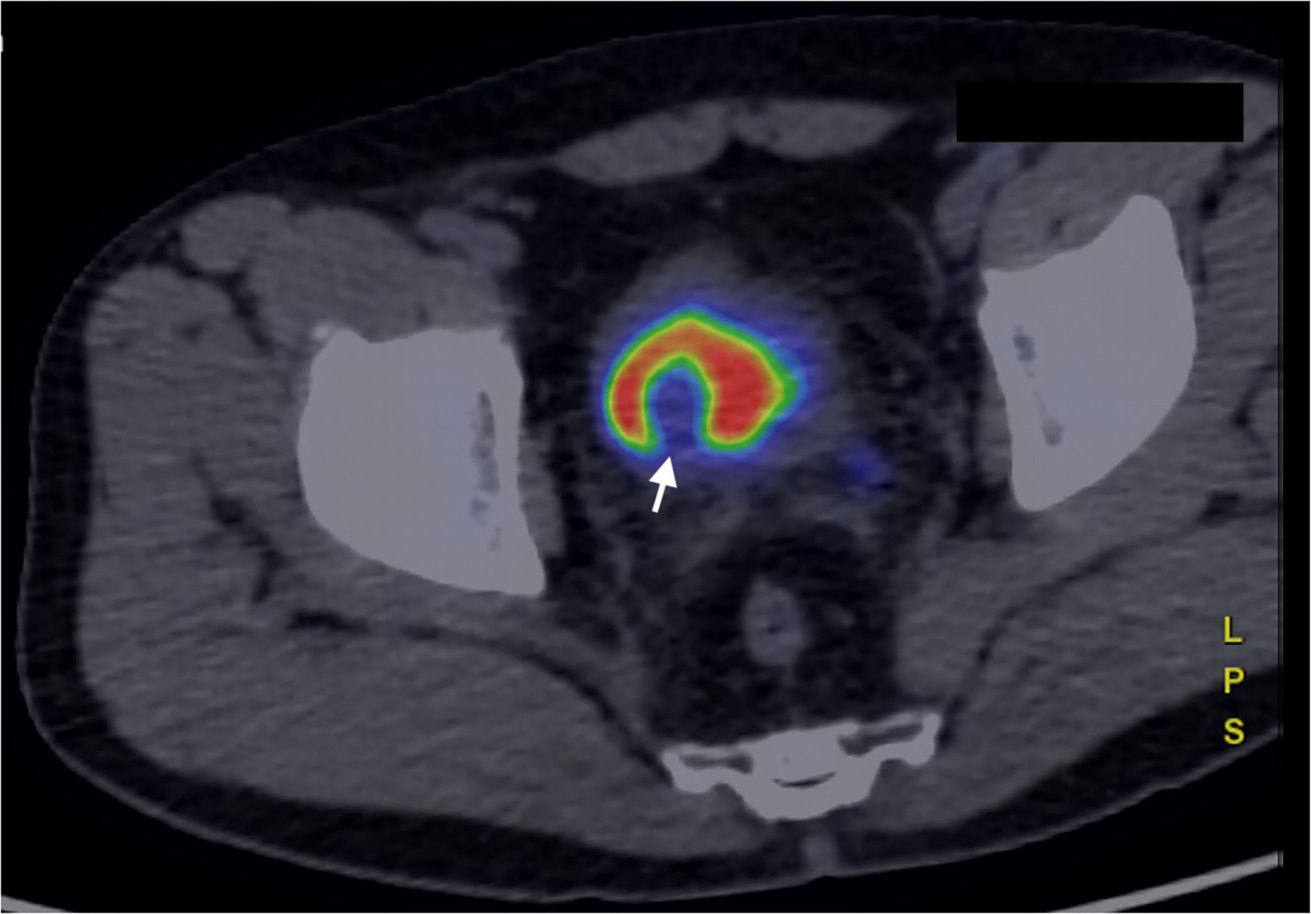
FDG PET/CT (image fusion technique) acquired in a late phase, 60 min after administration of the ^18^FDG. The bladder lesion is characterized by complete washout of the ^18^FDG, that collects in the urine, within the bladder (white arrow).

Nuclear medicine studies were thus performed to confirm the suspicion and to decide the best therapeutical workflow. PET/CT images where acquired with a field of view (FOV) extended from the vertex to the roots of the lower limbs, the slice thickness was 3.27 mm. In particular, FDG PET showed an early intense FDG uptake with a complete washout after 60 min (Figures 3, 4). This radio-nuclear pattern, along with the previous radiological findings, were consistent for Bladder Paraganglioma. Additionally, FDG PET did not show any further pathological radionuclide uptake.

To determine whether the described lesion was a functioning or a non-functioning BPG, urinary and blood metanephrines were evaluated, with values consistent for non-functioning tumor.

After proper premedication with α-blockers drugs, the patient underwent transurethral resection (TURB) and pathological report confirmed the clinical-radiological suspicion.

Four and twelve months follow up with CT scan and 1 year follow up with FDG PET/CT didn't show any signs of tumor relapse.

## Discussion

Although bladder cancer is one of the most frequent neoplasms, BPG accounts only for 0.06% of the totality of bladder tumors (19, 20). Despite being very uncommon, BPG must be included in the differential diagnosis, mostly because its management should be different from that of urothelial carcinoma, considering the high risk of hypertensive crisis after biopsy and during surgical resection. Whilst cystoscopy and urinary cytology are considered the main tools for urothelial carcinoma identification diagnosis, radiology and nuclear medicine are considered essential for telling BPG apart (21, 22).

In this case, the patient presented with hematuria and urinary retention, symptoms non-specific for BPG but more suggestive for aggressive urothelial carcinoma. A preliminary CT-scan with a delayed urographic phase suggested the presence either of a vascular malformation or a hypervascular solid tumor, situated on the bladder dome, where 40% of PGLs are usually found and could give a suspicion (8–10).

This clinical case shows three important elements to consider during the diagnostical workflow in bladder cancer and in particular BPGs.

First of all, the combination of radiological and nuclear medicine imaging techniques with laboratory tests (urinary metanephrines) led to the diagnosis of non-functioning BPG, granting the patient the chance to avoid a radical cystectomy while undergoing a curative, less invasive, procedure (TURB).

Secondly, ultrasound confirms to be the most useful first-level, cost-effective and non-invasive approach that can however provide some extra-information integrating more complex, second level, imaging techniques such as CT-scan and that might therefore help to choose the following steps.

Lastly, while ^68^Ga-DOTANOC PET/CT is considered the best nuclear medicine exam for the identification and diagnosis of BPGs yet, many centers would not have the chance to routinely perform this kind of exam. In this case dynamic FDG PET-CT (by far more accessible and commonly performed) might integrate the radiological techniques and confirm the suspicion of paraganglioma.

## Conclusion

In the presented case, taking into account the impossibility to perform ^68^Ga-DOTANOC PET/CT or ^123^I-MIBG SPECT/CT, accurate interdisciplinary communication and integration of radiological imaging with dynamic FDG PET/CT ensured an early diagnosis and prompt treatment of urinary bladder paraganglioma.

## Data availability statement

The original contributions presented in the study are included in the article/supplementary material, further inquiries can be directed to the corresponding author.

## Author contributions

MTT and AC: data collection and drafting the article. CG: conception of the work and critical revision of the article. FC, BC, LB, RS, EB, and RG: final approval of the version to be published. All authors contributed to the article and approved the submitted version.

## Funding

The work reported in this publication was funded by the Italian Ministry of Health, RC-2022-2773395.

## Conflict of interest

The authors declare that the research was conducted in the absence of any commercial or financial relationships that could be construed as a potential conflict of interest.

## Publisher's note

All claims expressed in this article are solely those of the authors and do not necessarily represent those of their affiliated organizations, or those of the publisher, the editors and the reviewers. Any product that may be evaluated in this article, or claim that may be made by its manufacturer, is not guaranteed or endorsed by the publisher.
